# Effects of beta-hydroxy-beta-methylbutyrate (HMB) on exercise performance and body composition across varying levels of age, sex, and training experience: A review

**DOI:** 10.1186/1743-7075-5-1

**Published:** 2008-01-03

**Authors:** Gabriel J Wilson, Jacob M Wilson, Anssi H Manninen

**Affiliations:** 1 Division of Nutritional Sciences, University of Illinois, Urbana, Illinois, USA; 2 Department of Nutrition, Food and Exercise Science, Florida State University, Tallahassee, Florida, USA; 3 Manninen Nutraceuticals Oy, Oulu, Finland

## Abstract

The leucine metabolite beta-hydroxy-beta-methylbutyrate (HMB) has been extensively used as an ergogenic aid; particularly among bodybuilders and strength/power athletes, who use it to promote exercise performance and skeletal muscle hypertrophy. While numerous studies have supported the efficacy of HMB in exercise and clinical conditions, there have been a number of conflicting results. Therefore, the first purpose of this paper will be to provide an in depth and objective analysis of HMB research. Special care is taken to present critical details of each study in an attempt to both examine the effectiveness of HMB as well as explain possible reasons for conflicting results seen in the literature. Within this analysis, moderator variables such as age, training experience, various states of muscle catabolism, and optimal dosages of HMB are discussed. The validity of dependent measurements, clustering of data, and a conflict of interest bias will also be analyzed. A second purpose of this paper is to provide a comprehensive discussion on possible mechanisms, which HMB may operate through. Currently, the most readily discussed mechanism has been attributed to HMB as a precursor to the rate limiting enzyme to cholesterol synthesis HMG-coenzyme A reductase. However, an increase in research has been directed towards possible proteolytic pathways HMB may operate through. Evidence from cachectic cancer studies suggests that HMB may inhibit the ubiquitin-proteasome proteolytic pathway responsible for the specific degradation of intracellular proteins. HMB may also directly stimulate protein synthesis, through an mTOR dependent mechanism. Finally, special care has been taken to provide future research implications.

## Introduction

The branched chain amino acids (BCAAs) leucine, isoleucine, and valine make up more than one third of muscle protein [1]. Of these, the most investigated BCAA is leucine, due to its broad effects, including: important roles in protein metabolism [2,3], glucose homeostasis [4], insulin action [5], and recovery from exercise [6]. For 35 years now, it has been known that leucine has anti-catabolic properties [7]. The mechanism by which this occurs has not been clearly established; however, it has been hypothesized that the metabolite of leucine, a-ketoisocaproate (KIC) may contribute to these results. To elaborate, when ingested, leucine is transaminated into KIC [8], which appears to decrease muscle breakdown [9-11]. However, there are conflicting studies that suggest that these effects may only take place under states of severe stress such as starvation [12] or in severe burn victims [13]. It also appears that the amount of BCAA supplementation affects its benefits. Supplementing with 16 grams of BCAAs resulted in several specific ergogenic benefits [14], while supplementing with 3 grams in a similar study did not [15]. Leucine is only partly converted into specific metabolites such as KIC, suggesting that this dose dependent response is in part dependent on a high enough provision of substrate to produce the metabolites necessary to optimize leucine's ergogenic effects. Further evidence has indicated that leucine's effects on protein degradation are prevented when transamination is inhibited [16].

After leucine is metabolized to KIC, KIC is either metabolized into isovaleryl-CoA by the enzyme a-ketoacid dehydrogenase in the mitochondria, or into beta-hydroxy-beta-methylbutyrate (HMB) in the cytosol, by the enzyme a-ketoisocaproate dioxygenase [8]. The majority of KIC is converted into isovaleryl-CoA, while under normal conditions; approximately 5% of leucine is metabolized into HMB [8]. In perspective an individual would need to consume 60 g of leucine in order to obtain 3 g of HMB, which is the most frequently administered dosage for HMB in studies.

A number of studies have indicated that HMB supplementation may elicit several ergogenic benefits, including anti-catabolic [17], anabolic [18], and lipolytic effects [19], among others [20]. Thus, it has been suggested that HMB may partly be responsible for the benefits of leucine supplementation. Given that HMB is a metabolite of leucine, and can be consumed through both plant and animal foods such as grapefruit and catfish, it has been credited as a dietary supplement [21-23]. Supplemental HMB is commercially available as calcium HMB monohydrate under 5 U.S. patents: 5,348,979 (a method for improving nitrogen retention), 5,360,631 (a method decreasing low-density and total cholesterol), 6,103,764 (a method for increasing aerobic capacity of muscle), 4,992,470 (method of enhancing immune response), and 6,291,525 (method for improving a human's perception of his emotional state), and 6,031,000 (composition comprising β-hydroxy-β-methylbutyric acid and at least one amino acid and methods of use).

HMB has been extensively used as an ergogenic aid; particularly among bodybuilders and strength/power athletes, who use it to promote exercise performance and skeletal muscle hypertrophy [24]. While numerous studies have supported the efficacy of HMB in exercise and clinical conditions [25-27], there have been a number of conflicting results. Therefore, the first purpose of this paper will be to provide an in depth and objective analysis of HMB literature. Special care is taken to present critical details of each study in an attempt to explain possible reasons for conflicting results seen. The second purpose of this paper is to provide an in depth analysis of possible mechanisms that HMB may exert its effects. Areas which will be considered include HMB's capacity to prevent muscle damage, lower protein degradation, and directly stimulate protein synthesis.

### Dependent measures used to study HMB supplementation

Several dependent measures have been utilized to study the effects of HMB supplementation. These include performance measures relating to dynamic [28], isometric and isokinetic strength [19], as well as functionality exercises in the elderly [29]. Other measurements include questionnaires to measure the extent of delayed-onset muscular soreness (DOMS) [30]; and various markers of health including blood pressure, cholesterol, and immune cell function [31]. Finally, given that HMB is generally considered to be an anticatabolic agent, markers of muscle damage are also commonly analyzed [32]. During physical exercise, muscle fiber disruption and subsequent increased permeability allow leakage of creatine kinase (CK), lactate dehydrogenase (LDH), and 3-methylhistidine (3-MH) into plasma, which is inferred to reflect the extent of incurred muscle damage [17,33,34]. Recently, HMB's possible effects on protein synthesis as assessed through uptake of radiolabeled phenylalanine has also been examined [35].

### Studies supporting the efficacy of HMB supplementation

The following sections will analyze various studies which support the efficacy of HMB supplementation. Independent variables analyzed will include training experience, age, and various catabolic states.

### The efficacy of HMB supplementation for untrained participants

Nissen et al. [36] examined the effects of HMB on muscle metabolism and performance during resistance-exercise in two experiments in healthy untrained males. Participants in the first experiment ingested 0, 1.5, or 3 g of HMB daily, while weight lifting 3 days per week for 3 weeks. Two dosages of HMB (0 or 3 g) were used in experiment two, with weight training occurring 2–3 hours daily for 7 weeks. Results from experiment 1 found that HMB decreased plasma markers of muscle damage (CK) and protein degradation (3-MH) in a dose dependent response with a range of 20–60%. Total weight lifted also increased in a dose dependent manner (8, 13, and 18.4% for 0, 1.5, and 3 grams of HMB, respectively). Finally, lean body mass (LBM) increased with each increment increase in HMB ingestion (0.4, 0.8, and 1.2 kg of LBM gain for 0, 1.5, and 3.0 grams of HMB, respectively).

In the second experiment, LBM was significantly increased with the HMB supplemented participants compared to the non-supplemented participants at weeks 2 and 4–6 with no further differences seen during the final week (week 7). Diminished improvements in LBM during the final week of training may be due to accommodation of the participants to the training stimulus. Participants ingesting HMB increased their 1 repetition maximum (1-RM) bench press by an average of 15 pounds, compared to a 5-pound increase in the non-supplemented group.

In two acute studies, Van Someren et al. [32,37] examined the effects of 3 grams of HMB and 0.3 grams of KIC on indices of muscle damage following a single bout of eccentric exercise in untrained male participants. Measurements were taken at 1, 24, and 72 hours post-exercise. Both studies indicated that DOMS, plasma CK, decrements in 1-RM bicep curling strength, and decreased range of motion (ROM) were reduced by HMB.

Gallagher et al. [19] investigated the effects of 0, 3, or 6 grams HMB supplementation in 37 untrained men on strength and LBM during 8 weeks of resistance training with 10 different resistance exercises, performed 3 times per week at 80% of the participant's 1-RM. While results found no significant differences between conditions in 1-RM or body fat mass after 8 weeks of training, the HMB supplemented conditions lowered plasma CK, and increased peak isometric torque, various isokinetic torque values and LBM to a greater extent than placebo. No differences were found between 3 or 6 gram conditions.

Jowko et al. [18] investigated whether creatine and HMB act by similar or different mechanisms in 40 participants, who resistance trained and consumed creatine, HMB, or creatine and HMB for a total of 3 weeks. Results found that HMB, creatine, and the combination group gained .39, .92, and 1.54 kg of LBM, respectively, above the placebo. The total amount of weight lifted increased above the placebo on all exercises combined was 37.5, 39.1, and 51.9 kg for HMB, creatine, and the combination group, respectively. Both HMB-supplemented conditions decreased CK, urine urea nitrogen, and plasma urea, while creatine supplementation alone did not decrease these markers. The apparent additive effects of these supplements indicate that creatine and HMB operate through separate mechanisms.

### The efficacy of HMB supplementation for experienced athletes

Several studies have found that HMB supplementation enhances LBM and indices of performance during resistance training, independent of training experience. Nissen et al. [28] investigated HMB supplementation on strength and body composition in trained and untrained males undergoing intense resistance training. Greater decreases in body fat and increases in LBM were found with HMB supplementation regardless of training status. Further, there was an overall 55% greater increase in bench press performance. Similarly, Panton et al. [38] examined the effects of HMB during resistance training in 36 women and 39 men, 20 to 40 years of age, with varying levels of training experience for 4 weeks. The HMB group decreased body fat to a greater extent (-1.1% *vs*. -.5%), and had greater increases in upper body strength (7.5 *vs*. 5.2 kg), and LBM (1.4 vs. .9 kg) than the placebo group, independent of training experience. Likewise, Thomson [39] found an increase in leg extension 1-RM relative to placebo (14.7% *vs*. 4.8%) after 9 weeks of strength training in 34 resistance trained men, while Neighbors et al. [40] reported that HMB decreased body fat and increased LBM in experienced football players.

Finally, Nissen and Sharp [41] performed a meta-analysis concerning dietary supplements postulated to augment lean mass and strength gains during resistance training. Studies between the years 1967 and 2001 were included, if they met their strict experimental criteria, including at least 3 weeks of resistance training, 2 or more times per week. Over 250 supplements were analyzed; however, only 6 met their criterion. Results found that only creatine (18 studies) and HMB (9 studies including both trained and untrained participants) had sufficient data supporting their ability to enhance LBM and various indexes of performance. They found that HMB supplementation at 3 grams per day resulted in a net increase of .28% and 1.4% per week for LBM and strength gains, respectively.

The efficacy of HMB has also been replicated in measures of performance in experienced endurance athletes. Vukovich and Geri [42] investigated the effects of HMB supplementation on peak oxygen consumption (VO_2 _peak) and the onset of blood lactate accumulation (OBLA) in eight endurance-trained master-level competitive cyclists, with an average training volume of 300 miles per week. Participants performed a graded cycle ergometer test until exhaustion. All participants performed 3, 2-week supplementation protocols consisting of either 3 grams of HMB, leucine, or a placebo daily, while continuing their normal training volume. Results from the graded exercise test indicated that HMB increased the time to reach VO_2 _peak (8%), while leucine and the placebo did not. The VO_2 _at 2 mM of lactate (OBLA) increased with HMB (9.1%) and leucine (2.1%), but not with the placebo. Likewise, Vukovich and Adams [43] found that 2 weeks of HMB supplementation in experienced cyclists increased both VO2 peak and the time to reach VO_2 _peak, while supplementation with leucine or a placebo did not change these measurements.

Knitter et al. [17] examined the effects of 3 grams of HMB or a placebo on muscle damage during a 20 km run in 16 experienced male and female long distance runners. Results showed a decrease in LDH and CK levels with the HMB supplemented participants compared to the non-supplemented participants. These results agreed with Byrd et al. [44] who found that HMB or HMB combined with creatine equally decreased the rise in muscle soreness following a 30 minute downhill run in 28 young, active males; while the creatine only and placebo group did not.

### The efficacy of HMB supplementation in the elderly

Several studies have examined if the ergogenic benefits of HMB supplementation can be generalized to the elderly. Vukovich et al. [29] showed that HMB supplementation in 31 untrained, elderly men and women during an 8 week resistance training program resulted in increased body fat lost (-.66 *vs*. -.03 %), and greater upper (13% *vs*. 11%) and lower body strength (13 % *vs*. 7 %) in the HMB condition than the placebo.

Flakoll et al. [45] performed an experiment to determine whether arginine and lysine, which may increase protein synthesis, and HMB, which may decrease protein breakdown, could blunt sarcopenia. Fifty elderly women (M = 76.7 y) consumed a placebo or 2 grams of HMB, 5 grams of arginine, and 1.5 grams of lysine daily. After 12 weeks, there was a 17% increase in the "get-up-and-go" test in the experimental group but no change in the placebo group. There were also increases in limb circumference, leg strength, handgrip strength, and a 20% increase in protein synthesis over a 24-hour free-living period relative to the placebo. Positive trends in fat-free mass gains (p = 0.08) were also detected.

Vukovich et al. [46] investigated the efficacy of 3 grams of HMB supplementation daily, for 8 weeks, in a group of 70-year old individuals, exercising 2 days per week. Results indicated greater fat loss (-4.07 *vs*. .31 %) and greater strength gains (17.2 *vs*. 8.3 %) during the first 4 weeks of supplementation in the HMB supplemented condition versus the placebo. Panton et al. [47] investigated the effects of HMB on muscle strength and functional ability in 35 70-y old male and female individuals, who participated in a 12-week resistance-training program. Prior to and post training, changes in leg extension and chest press capacity; time to get out of a chair, walk 6.6 meters, turn around, walk back to the chair, and sit down (GUG); and time to walk 15.2 m at their regular stride length were measured. No significant differences were found between leg extension and chest press strength, or walking time between the HMB and placebo groups. However, GUG significantly (p < .05) improved over the placebo with HMB supplementation.

### The efficacy of HMB supplementation during states of severe muscle catabolism

As a purported anti-catabolic agent, HMB supplementation has been examined under various muscle wasting situations. Soares et al. [48] showed that HMB supplementation during hind limb immobilization of adult mice resulted in less fiber damage, and greater muscle fiber diameter (+6.9%). Consistent with these results, studies have found that HMB supplementation decreases performance decrements associated with bed rest [49,50]. Cohen [51] investigated the effects of HMB supplementation on changes in body composition during positive and negative energy balances. Results found that HMB supplementation maintained LBM to a greater extent than placebo while in a negative energy balance. This is consistent with similar studies on the effects of amino acids and their metabolites during negative energy balance [52-54].

Studies also indicate that HMB can reduce muscle loss associated with diseases such as auto immunodeficiency syndrome (AIDS) [55,56], and cachectic cancerous conditions [35]. Collectively, these results led Alon et al. [57] in a review on HMB to suggest that "continuing research in HMB treatment of patients with advanced-stage disease may potentially uncover methods to increase strength and immunity and thus improve chances of survival (p.g. 14)."

### Additional studies which support the efficacy of HMB supplementation

The following section will analyze remaining studies found which support the efficacy of HMB supplementation, but did not fit into the aforementioned categories. Coelho and Carvalho [52] sought to determine if HMB would be beneficial to 12 males between the ages of 50 and 72, with hypercholesterolemia, who exercised five times per week for 4 weeks with a combination of endurance and resistance training. Results showed that low density lipoprotein cholesterol (LDL-C) levels lowered from 172 to 123 mg/dl, LBM increased 6%, and performance improved in every lift including leg presses (+1.8 kg), rear lat pull-downs (+1.5 kg), and biceps curls (1.5 kg) in the HMB group. The placebo group showed no differences in cholesterol levels, but did improve performance in the leg press (+1.3 kg) and rear lat pull-downs (+ 1.8 kg).

There is also evidence to suggest that HMB increases fat oxidation in muscle cells [58,59]. Lastly, several animal studies have found benefits from HMB including decreased body fat [58], blood cholesterol [60], and muscle proteolysis [61,62]. HMB supplementation in the absence of exercise, however, does not appear to have ergogenic benefits in healthy individuals [63], suggesting that HMB supplementation may only be effective with increases in muscle catabolism.

### Studies which do not support the Efficacy of HMB supplementation

A number of studies conflict with research that supports the efficacy of HMB supplementation. The following section will analyze these studies.

Kreider et al. [64] used 40 experienced (M = 5 y) resistance trained athletes who averaged 7 hours of training per week for 28 days, while supplementing with 0, 3, or 6 g of HMB daily. Participants were not monitored, but instead, were instructed to maintain their normal training programs during the experiment and record their training volume before and after the experiment in a log. Consistent with this, no differences were found in training volume performed before and after supplementation with HMB; while training intensity was not reported. Results showed no significant decrease in markers of muscle damage, fat mass, increased LBM or 1 RM performance in any of the lifts measured in the placebo or HMB supplemented conditions.

Slater et al. [65] had experienced resistance trained males (M = 2 y) consume 3 g of HMB or a placebo for 6 weeks while performing 2–3 sessions weekly of compound movements (e.g. leg press, chins, bench press), for a total of 24–32 sets, at a training intensity of 4–6 repetitions. The training intervention significantly increased lean body mass and total strength gains, but did not increase any of the individual lifts. HMB supplementation had no significant effect on LBM or strength or biochemical markers of muscle damage.

Paddon-Jones et al. [66] examined the effects of HMB on symptoms of muscle damage following a bout of eccentric exercise. Participants were non-resistance trained males, who consumed HMB or a placebo, 6 days prior to and after a bout of 24 maximal isokinetic eccentric contractions of the elbow flexors. Muscle soreness was measured using a 10 point visual analogue scale, with a response range from no soreness to extreme soreness. Arm girth was measured with a metal tape measure, and muscle torque was also measured at 15 minutes and 1, 2, 3, 7, and 10 days post exercise. The exercise bout significantly (p < .05) increased muscle soreness, peaking at a score of 7; but there was no significant difference between conditions in muscle soreness, ROM, or elbow flexor strength. In a similar experiment, Jennifer et al. [67] found no significant difference in ROM or DOMS from HMB supplementation.

O'Connor and Crowe [20] investigated the effects of HMB or HMB and creatine supplementation in elite, male rugby players. Testing involved a multistage fitness test to determine aerobic power and a 60 second maximal cycle test to determine anaerobic capacity. No significant differences were showed in either condition for any of the measures taken.

Jack et al. [68] examined the effects of daily HMB supplementation on muscular strength (bench press, squats, and power cleans) and body composition (body weight and body fat) among elite collegiate football players who trained 20 hours per week for 4 weeks. Results found no significant benefits from HMB in bench press, squats, or power clean performance, and no significant changes in body composition. The lack of improvement overall from this program lead the authors to conclude that, "...subjects may have been over trained. The volume of exercise in this study was higher than most other HMB-supplementation studies. Although HMB may be most effective when increasing training volume or intensity, the extremely high total training load may have attenuated the potential effectiveness of HMB to reduce muscle damage or protein breakdown."

Similar to the aforementioned experiment, Hoffman et al. [69] investigated the effects of HMB on power performance (using the Wingate anaerobic power test), indices of muscle damage, and stress in 26 collegiate football players, during a 10-day training camp. Results found no significant differences among conditions in markers of stress (testosterone/cortisol ratio) and markers of muscle damage (myoglobin and CK); finally, there was no significant increase in performance in either condition pre to post test.

Kreider et al. [70] examined the effects of 3 grams of HMB on Division 1-A College Football players over 4 weeks of training. Training was supervised, and consisted of 5 hours per week of resistance training with movements such as bench press, shoulder press, and squats. Lifts were prescribed at 1–3 sets, 2–8 reps, at 60–90% intensities. Football sprints and agility drills were also performed 3 hours per week. Training significantly (p < .05) increased total body mass, LBM, biochemical markers of muscle damage, and decreased body fat percentage; however, there were no significant differences between conditions in any of these variables. Lastly, there was no significant difference between conditions in combined lifting volume, or repetitive sprint performance.

### Possible explanations for conflicting results

It is critical to analyze possible explanations for conflicting results. To begin, in practically any investigation, the possibility of obtaining contradictory results is high, based on the inherent noise (variability) found across human participants [71]. The effects of variability in humans on behavioral measures was first quantitatively analyzed by Clark Hull in the 1940s [72]. Hull suggested that performance was determined by seven components such as internal drive states (e.g., motivation) that were variable in nature. Since Hull, numerous studies have confirmed that results in human performance are not only affected by physiological states, which are the primary target of HMB, but also through numerous other variables including: the participant's social milieu (e.g., social facilitation/debilitation) [73], motivations (intrinsic and extrinsic) [74], self-confidence [73], and current emotive states [75]. According to Schmidt and Lee [71], the most effective way to 'tease' out variable behavior is through obtaining adequate sample sizes. Unfortunately, it is often difficult for scientists to obtain large samples [76], as indicated in a number of studies conducted on HMB in which sample sizes are comprised of 8 or fewer participants [32,42,43,67], while the results obtained are generalized to millions of people worldwide. Scientists are also often limited to biased sampling, such as sampling by availability [77,78] and convenience [76]. Thus, contradictory studies should not be surprising in biological research–rather, they should be expected.

A number of qualitative and quantitative solutions exist to deal with this problem. Qualitatively, comprehensive reviews are able to synthesize numerous studies in order to find trends in the literature. Quantitatively the effect sizes from hundreds of subjects across several studies can be combined.

Other problems that occur lie in the validity of the testing conditions. As will be discussed, certain tests may not serve as a valid means of measuring what HMB supplementation is purported to effect. A second problem that stems from invalid measurements is that the conclusions drawn from them may also be invalid. The following section details specific examples of methodological problems, which may partly explain contradictory results found in HMB-related literature.

### The efficacy of HMB in trained athletes

While several studies have found benefits from HMB in trained athletes [38-44], a number of studies have not [64,68,69], leading some authors to conclude that HMB supplementation may not be effective in trained individuals [64,69]. Bloomer and Goldfarb [79] in a review on sports supplements concluded that, "although it may be somewhat reasonable to consider this nutrient [HMB] during the initial stages of training, regular trainees may not benefit much from its use." Similarly, Hoffman et al. [69] posited that "if HMB supplementation has any ergogenic benefit in attenuating muscle damage, it is likely to be most effective in the untrained population where the potential for muscle damage to occur during exercise is greatest."

Three possible explanations for the discrepancies found in studies researching trained individuals will be discussed. One was described by Hoffman et al. [69] and suggests that the benefits of supplementing with HMB may be maximized when muscular damage is heightened. For example, Nissen et al. [63] investigated the effect of supplementing with HMB on body composition and performance in both exercising and non-exercising women. Results showed HMB supplementation increased LBM, fat loss, and performance in women who did exercise, but not for women who did not exercise. In trained individuals, however, a stimulus must be substantially greater than untrained individuals to cause significant disruption [80]. Of particular interest to HMB research is the finding that the amount of muscular damage elicited by the same eccentric bout of exercise decreases by the second bout [81] (This concept will be discussed in further detail under the section on mechanisms of HMB action). These findings highlight the need for variability in training programs; particularly, in elite athletes. This concept can be applied to the Kreider et al. [64] study. In this study, the athletes were not monitored, but rather, instructed to maintain their same training volume which they had previously performed prior to supplementation with HMB. Since no significant decreases in markers of muscle damage, fat mass, increased LBM or 1 RM performance in lifts measured were found in any of the examined conditions, this would suggest that athletes were accommodated to the training stimulus. To properly examine the efficacy of HMB, future studies are encouraged to design a periodized, and monitored, strength program, which the athletes are not accommodated to, that increases performance across conditions.

A second explanation for conflicting results seen in experienced athletes consists of methodologies which contain a lack of specificity between training and testing conditions. As a brief review, over a century of evidence has indicated that motor tasks are highly specific in nature and have little transfer to other tasks (for excellent reviews, see references [71,82-84]). One of numerous examples of the extent of the specificity principle was identified by Rivenes and Sawyer [85] who calculated the amount of shared variance (r2) from over 1740 intercorrelations between 60 motor tasks commonly used to examine strength, flexibility, and power in 204 males of the US Navy Academy. These investigators found an average of only 7 % commonality between tasks, indicating that strength, flexibility, and power are task-specific.

An understanding of the specificity principle can be applied to a study by O'Connor and Crowe [20]. As previously discussed, the rugby players examined consumed HMB during the course of their normal season, while testing involved a multistage fitness examination to determine aerobic power and a 60 second maximal cycle test to determine anaerobic capacity. No significant increases in the tests were shown in the HMB or placebo conditions. The lack of positive results may be explained by non-specific testing criteria relative to Rugby practice. For example, cycling is not an intrinsic part of Rugby playing conditions, while multistage fitness testing has been demonstrated to have low shared variance with other tasks [85-87].

Similarly, one measurement Hoffman et al. [69] used to asses the efficacy of HMB in football players, was performance in the Wingate anaerobic power test. As would be predicted by the specificity principle, results found no significant increase in performance in either condition. A third variable which may confound results concerns the time periods used in studies to analyze advanced athletes. For example, Hoffman et al. [69] analyzed HMB during a short 10-day training camp. Slater and Jenkins [88] suggested that (p.g. 112):

"It may be that for highly trained individuals, 4 weeks of HMB supplementation is an inadequate time frame to allow adaptations unique to HMB supplementation to be identified. Studies involving longer periods of supplementation, as used in some of the trials with untrained volunteers, are needed to address this issue."

It is interesting to note that all but 2 acute studies discussed in this manuscript [66,67], were found to support the efficacy of HMB supplementation in untrained participants. Therefore, it would appear that the focus of HMB research should be on trained populations.

### Other possible adjustments to future methodologies

A further problem suggested by the current authors is the dosage of HMB administered. Currently, it is advised to have 3 grams of HMB per day, spread into 3 equal dosages. But few studies have actually investigated the optimal dosage and optimal frequency of this supplement. Optimal dosages will be discussed further on in this manuscript. An additional problem is that while there are numerous dependent measures used to determine the efficacy of HMB, few studies have actually fully utilized these measurements.

In summary, while various studies in this review support the efficacy of HMB supplementation, several studies did not. These conflicting results may be partly attributed to variability in humans, inadequate sample sizes, and methodological issues including the specificity of testing conditions, cases of overtraining, elicitation of an inadequate training stimulus in experienced participants, limited dependent variables, and short duration experiments. Collectively, these results warrant further research on HMB supplementation while taking into account these various issues.

### Clustering as a proposed problem against the validity of HMB experiments

An argument sometimes proposed against the efficacy of HMB supplementation, is that the studies concerning this supplement tend to be conducted by similar authors (clustered). For example, Jacques et al. [89] noted that in the Nissen and Sharp [41] meta-analysis "the nine studies on HMB clustered around three unrelated groups of researchers (p.g. 2,180)."

The premise of the argument by Jacques et al. [89] was that unassociated studies might elicit different results than associated studies. Furthermore, it was proposed that associated studies might elicit similar results to each other, due to similar methodological techniques. However, this was not supported in the meta-analysis by Nissen and Sharp [41]. Their results indicated that the average effect size for the associated studies was .16, while the unassociated studies had a similar effect size of .14. Moreover, the results sharply varied among the associated studies, with the effect sizes ranging from .03–.43, leading Nissen and Sharp [89] to argue that, "With all effect sizes for the HMB studies being generally similar and the ranges of the associated studies including the unassociated studies, it seems unlikely that any source of systematic bias explains the difference. The small numerical differences are more likely to result from varying procedures, dosages, measurements, and subject variability (p.g. 2, 182)."

A further argument proposed against the validity of HMB investigations, is that the studies which have been done by authors who profit from HMB sales, are subject to bias, due to a conflict of interests, and therefore, may not be trustworthy [90]. However, this argument can be classified as an Ad Hominem Circumstantial argument, which is an argument suggesting that because someone may benefit by taking a certain stance, their evidence is therefore, invalid. While a bias may be cause for concern, it is unsubstantiated to conclude that the evidence presented by the party in question, is therefore, invalid, and untrustworthy.

Finally, under the assumption that this argument was correct, the attractiveness of science is that it is replicable and consequently self correcting. Therefore, if bias in studies conducted on HMB, led to erroneous results, others should not be able to find similar results in their experiments. To further validate experiments conducted on HMB, the current authors propose that an updated meta-analysis is needed, as the meta-analysis by Nissen and Sharp [41] was done on a small sample of studies (9) and was performed over 6 years ago. The meta-analysis will want to pay close attention to various moderator variables including, exercise modality, training loads, training experience, age, and several dependent measures such as markers of muscle damage, strength, and DOMS. Furthermore, an additional analysis of the range of authors and Universities, which performed these studies would be helpful, to further negate the issue of clustering.

### Safety and health benefits of HMB supplementation

Studies have found that people are consuming more than the recommended 3 g per day dosage of HMB [91]. Thus, it is imperative to analyze the safety of various dosages of HMB. Currently, studies have found no potential adverse side effects when supplementing with HMB in both humans consuming 3–6 grams daily [63,92-94] and animals consuming variable dosages [11,30,95-98]. In fact, no adverse effects have been seen in animals consuming enormous amounts of HMB, with a range between 8 and 5000 mg·kg-1·day-1 for a period of 1–16 weeks [60,63,99]. For a 200 pound man, that would be an upwards of 450 g of HMB per day.

Nissen et al. [36] performed an extensive two-part experiment to test the effects of HMB supplementation on muscle metabolism during resistance-exercise training. Results found no adverse side effects from HMB supplementation. Similarly, Matthew et al. [29] investigated whether 3 grams of HMB daily would benefit 70-y old adults. Results from this study also indicated no adverse effects from HMB supplementation. Thus, HMB appears to be safe when taken over several months, with 3–6 gram dosages in humans. Additionally, as will be shown below, HMB may actually be beneficial to various indexes of health.

Nissen, Sharp, and Panton [31] analyzed safety data from nine studies in which humans were fed 3 g of HMB per day. The studies were from 3 to 8 weeks in duration, and included both males and females, young and elderly, exercising and non-exercising participants. Results found that HMB did not negatively affect any indicator of tissue health or function. Further, HMB significantly (p < .05) improved one measurement of negative mood state. It was also found that HMB supplementation resulted in a net decrease in total cholesterol (5.8%), a decrease in systolic blood pressure (4.4 mm Hg), and a decrease in LDL-C (7.3%). However, HMB did not significantly lower LDL-C in subjects with accepted normative levels of cholesterol (< 200 mg/dl), suggesting that HMB is more effective at lowering LDL-C when cholesterol levels are high. Consistent with this, Coelho and Carvalho [52] found that HMB supplementation resulted in a significant (p < .05) decrease in LDL-C levels, going from 172 to 123 mg/dl, in individuals with hypercholesterolemia.

Gallagher et al. [93] investigated the effects of differing amounts of HMB (0, 3, and 6 g) on hematology and hepatic and renal function during 8 weeks of resistance training in untrained men. Results found no adverse effects from HMB supplementation on hepatic enzyme function, lipid profile, renal function, or the immune system. Evidence also suggests HMB may help the immune system and increases wound repair [98].

In summary, available evidence suggests that HMB supplementation is safe, and may potentially improve several markers of health.

### Optimal dosage of HMB supplementation

Most studies advise taking 3 grams of HMB daily for maximal benefit [[31,36,41,43], &[38]]. For instance, Nissen et al. [36] found that HMB in servings of 0, 1.5, and 3 grams improved performance in a dose dependent manner. However, it would have been interesting to observe the efficacy of higher dosages. More recently, Gallagher et al. [19] found that 6 grams of HMB did not improve LBM or strength gains over 3 grams.

To the current authors knowledge, this study by Gallagher et al. [19], along with the previously discussed Kreider et al. [64] study in trained individuals are the only studies to investigate the efficacy of dosages of HMB above 3 grams. Thus, it would be advisable that other scientists replicate these results under varying circumstances.

### Latency of peak of HMB concentration following ingestion

Vukovich et al. [100] investigated the digestion patterns of HMB, and the effect of glucose supplementation on HMB in 2 studies. Eight males consumed 1 g of HMB in study 1 and 3 g of HMB, or 3 g of HMB with 75 g of glucose in study 2. In the first study, plasma HMB peaked at 120 nmol/mL 2 hours after ingestion. Approximately 14% (0.14 g) of the HMB accumulated in the urine following ingestion of one g of HMB. In the second study, plasma HMB peaked at 487 nmol/mL 1 hour after ingestion of 3 g of HMB and was significantly lower at 352 nmol/mL 2 hours after ingestion of 3 g HMB and glucose. The authors suggested that the delay in peak concentrations of HMB coupled with research indicating slowed gastric emptying in response to increasing glucose concentrations, in solution suggests lowered plasma concentrations are at least partly due to gastric emptying. Based on the finding that glucose stimulated insulin secretion has been found to enhance skeletal muscle uptake of amino acid based substances it is possible that this hormone may have effected HMB concentrations through a similar mechanism. Currently the latter contention is speculative as the metabolic fate of HMB is unknown. Resolution of this uncertainty led the authors to suggest a further analysis using the hyperinsulinemic clamp technique. Approximately 29%(0.87 grams) of the ingested HMB accumulated in the urine following ingestion of HMB and glucose or HMB alone, with no significant differences between the two. In summary, plasma HMB peaks faster at 3 (60 minutes) vs. 1 (120 minutes) gram doses, and is delayed when consumed with glucose (60 vs. 120 minutes). Further, about 71 to 86% of consumed HMB is retained by the body, with a greater percentage of HMB being retained at 1 *vs*. 3 g dosages, independent of glucose consumption. Lastly, HMB has a half-life of approximately 2.5 h, and reaches baseline levels 9 hours after consumption.

Some authors have recommended that HMB should be standardized according to body weight. Using this framework, it is advised to have 38 mg/kg of body weight per day (equivalent to 17.3 mg/lb of body weight per day) [19].

In summary, when supplementing with HMB, current evidence suggests that 1 gram of HMB should be consumed 3 times per day, for a total of 3 g of HMB daily (or 38 mg/kg of LBM). However, clearly more studies are needed to determine the optimal dosage and frequency of HMB supplementation, and the overall efficacy of HMB supplementation as an ergogenic aid.

### Mechanisms of action proposed for HMB

**Figure 1:**
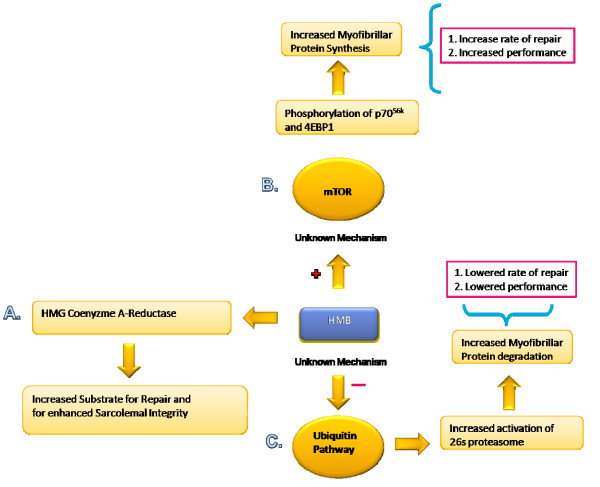
**Possible Mechanisms of HMB action**. HMBs proposed mechanisms of action include (A) Increased sarcolemal integrity via conversion to HMG-CoA reductase, (B) enhanced protein synthesis via the mTOR pathway and (C) depression of protein degradation through inhibition of the Ubiquitin pathway.

HMB's mechanisms of action are generally considered to operate through its capacity to stabilize the sarcolemma [94] and/or attenuate proteolytic pathways [35,101]. The role of HMB in stabilizing the sarcolemma is known as the Cholesterol Synthesis Hypothesis (CSH), while its antagonistic effects on proteolytic pathways appear to operate through the ubiquitin-proteasome dependent pathway (Ub-pathway). The following three sections will discuss (1) the CSH, (2) the effects of HMB on the Ub-pathway, and finally (3) how these mechanisms may interact to enhance both muscle tissue accretion and indexes of exercise performance (Figure 1)

### The Cholesterol Synthesis Hypothesis (CSH)

According to the CSH, a damaged muscle cell may lack the capacity to produce adequate amounts of cholesterol needed for various cellular functions, including the maintenance of sarcolemmal integrity [31]. This is particularly important in muscle tissue, which relies heavily on *de-novo *cholesterol synthesis [31]. Cholesterol is formed from Acetyl-CoA, in which the rate limiting step, catalyzed by the enzyme HMG CoA reductase, is the formation of the cholesterol precursor mevalonic acid from HMG-CoA. The majority of HMB is converted into HMG-CoA reductase [31,102]. Therefore, increased intramuscular HMB concentrations may provide readily available substrate for the synthesis of cholesterol needed to form and stabilize the sarcolemma [31,36].

Support for this hypothesis has come from the finding that the inhibition of cholesterol synthesis results in impaired muscular function [103], heightened muscular damage [104], and finally, muscle cell necrosis [105]. Paradoxically, studies have found that HMB is associated with lowered total and LDL-C levels, but only in cases of hypercholesterolemia (i.e., > 200 mg/dl) [31,52]. While no complete explanation has been offered, these effects may be related to the inclusion of calcium during HMB supplementation (100–200 mg per g of HMB). Research indicates that as low as a gram calcium supplementation lowers serum cholesterol concentrations through increasing bile acid excretion, leading to increased use of endogenous cholesterol from the liver for regenerative processes [106].

HMB's role in the production of mevalonic acid, may also serve other functions critical for muscle function [107]. Mevalonic acid, produced from HMG-CoA reductase is a precursor of coenzyme Q and dolichols [108], which are critical for myocyte proliferation [108]. Coenzyme Q also plays a major role in mitochondrial electron transport function [108,109].

### Possible onteraction of HMB with the ubiquitin-proteasome proteolysis dependent pathway

A number of new developments have occurred in the analysis of proteolytic pathways that HMB may interact with. The three major pathways through which proteolysis occurs are lysosomal, calcium activated calpain (CAC), and Ub-pathways [26,110]. Extra cellular proteins such as insulin receptors appear to be degraded through the lysosomal pathway [110], while the CAC system may have a role in the initial degradation of intracellular proteins [111]. Finally, the Ub-pathway appears to be responsible for specific intracellular protein degradation [26]. Increased activity of the Ub-pathway is common in conditions which elicit increased muscular proteolysis [112,113] including: antigravity conditions [114,115], cancer [26,101], limb immobilization [75], starvation [112], denervation [116], lowering of activity [117], and a variety of exercise conditions [118,119]. The efficacy of HMB has been demonstrated in both diseased [26,56] and exercise induced states of catabolism [36], indicating that it may operate through direct or indirect interference of the Ub-pathway.

Research on the effects of HMB on the Ub-pathway has been primarily conducted in the Tisdale lab, with notable studies conducted by Smith et al. [26,101], and more recently Baxter and colleagues [35]. Smith et al. [101] investigated the effects of HMB supplementation in cachexic tumour bearing mice. The study found that HMB increased muscle wet weight of the gastrocnemius, and lowered protein degradation relative to control animals. This was associated with a decrease in activity and expression of the Ub-pathway. Intriguingly enough, it was found that HMB supplementation also increased protein synthesis in the gastrocnemius.

To further isolate possible mechanisms involved in proteolytic pathway depression, Smith et al. [26] administered HMB to murine myotubes exposed to Proteolysis-Inducing Factor (PIF), which is associated with the up-regulation of the Ub-pathway. The increases in proteolysis through PIF administration were completely attenuated by HMB. These findings were accompanied by a decrease in the activity of protein kinase C and accumulation of nuclear factor-kappa B, which are critical components in PIF up-regulation of the Ub-pathway.

More recently Baxter et al. [35] investigated a possible mechanism by which HMB might stimulate protein synthesis. Because HMB is a metabolite of leucine, Baxter et al. [35] examined if HMB was able to activate protein synthesis through a similar mechanism as leucine. Leucine appears to stimulate protein synthesis through activation of mammalian target of rapamysin (mTOR) [120-122], a protein kinase indicated to up-regulate protein synthesis at the level of translation initiation [121].

Baxter et al. [35] utilized a similar protocol to the Smith et al. [101] study. However, to examine if HMB was operating through an mTOR dependent mechanism, rapamycin, a specific inhibitor of mTOR, was administered. HMB supplementation attenuated muscle tissue loss, which was partly attributed to an increase in protein synthesis relative to the control mice. However, Rapamycin attenuated the increase in protein synthesis, suggesting that HMB is operating either directly or indirectly through an mTOR-specific mechanism.

The role of HMB in attenuating the Ub-pathway in exercise induced proteolysis remains to be directly investigated. What studies do indicate, however, is that exercise appears to be associated with a three phase Ub-pathway response (for a review, see reference [117]). Phase one begins immediately following initiation of exercise and transiently (minutes to hours) increases the conjugation of Ub to substrate proteins [117]. This phase reverses after exercise has ended [99]. Phase 2, occurring 6–24 hours following exercise involves an increased expression of the Ub-pathway and is thought to be involved in remodeling of damaged muscle tissue [117]. Finally phase three occurs days to weeks after exercise and is associated with a return of Ub-proteasome expression to baseline levels [117]. Results indicate that amino acid supplements delivered prior to and after exercise markedly increases protein balance, and that this increase is associated with greater blood flow to the muscle tissue (for a review, see reference [3]). Future research implications involve studying the effects of the timing of HMB ingestion relative to exercise. If HMB attenuates the Ub-pathway, then its administration prior to exercise may specifically decrease the phase 1 Ub-proteasome response, while post exercise feedings may have specific effects for the phase 2 response.

Lastly, current research indicates that when a given training stimulus remains similar that the Ub-pathway response lowers with each successive exercise session [123]. If HMB is operating through the Ub-pathway to decrease muscle damage during exercise, then this finding reinforces the importance of incorporating variability during training in HMB experiments; particularly in experienced athletes who are more resistant to muscle damage.

### Applications of HMB mechanisms to indices of performance and lean mass

This section addresses how HMB's proposed mechanisms of action interact with and explain improvements in indexes of human performance and body composition. Increased protein accretion is a function of the difference between protein synthesis and protein degradation [25]. As indicated above, studies have shown that HMB may affect both functions [35], thereby increasing the ratio of protein synthesis to degradation [26], with a subsequent positive change in LBM. Changes in strength are largely due to neurological adaptations early in practice (changes in motor unit recruitment, asynchronous to synchronous contractions, etc.), while increases in lean muscle mass, which increases the capacity off the body to produce force, accounts for a greater percentage of strength gains later on [84]. Currently, the ability of HMB to increase indices of strength have been attributed to the changes observed in lean mass. However, research has not examined possible neurological adaptations facilitated by HMB supplementation.

HMB's effects on performance across time has been an area of interest. One study [36] indicated HMB was effective early in a training intervention (< 6 weeks), with lower benefits seen in the latter part of the intervention (week 7). Evidence from Willoughy et al. [123] suggests that Ub expression is lowered when participants are exposed to repeated bouts of similar training stimuli. If HMB is operating through the Ub-pathway then future studies will want to correlate Ub-expression with HMBs effectiveness. Ub may also serve as a dependent measure for the effectiveness of a stimulus to elicit enough disruption in athletes for HMB to be effective.

As discussed, some studies have indicated that HMB may increase body fat loss. For instance, Jack et al. [68] found that HMB increased beta-oxidation of the fatty acid palmitate by 30%. If HMB does lower body fat, these findings may be related to HMB's role in preventing the breakdown or stimulating the synthesis of proteins associated with the oxidative system. For instance, decreases in the breakdown of mitochondria, or increases in its synthesis, would potentially elevate an individual's capacity to metabolize fat. Evidence suggests that the success of fat loss interventions, which include exercise are associated with increased mitochondrial content and size [124]. HMB may also influence mitochondrial function through an increase in Coenzyme Q. However, to date no direct studies have investigated this proposal. Finally, increased muscle mass from HMB supplementation could increase participants metabolic rate, effectively increasing fat oxidation.

HMB's possible effects on sparing or enhancing the function of oxidative organelles, has received support from studies indicating that it can improve performance in exercise highly reliant on the oxidative system [42]. During HMB supplementation participants demonstrate a higher OBLA and VO_2 _peak [42]. The primary mechanism to clear lactic acid is through oxidation [42]; therefore, these results may be explained through increased mitochondrial content, size, or functional changes. Higher VO_2 _peaks may also be attributed to increased muscle tissue accretion [42].

Studies have indicated that HMB may lower blood pressure [31]. These effects may partly be attributed to the inclusion of calcium, [125] as the degree to which HMB lowers blood pressure is not much greater than what calcium normally does by itself [31]. Lastly, HMB appears to increase immune function in animals [126]. For example, HMB exposure increases macrophage proliferation and functionality as indicated through phagocytosis. HMB may exert its effects through an mTOR related mechanism, as this kinase is critical for lymphocyte proliferation [127].

### Implications for future research

The first purpose of this paper was to provide an in depth and objective analysis of HMB research. A reflection on the data discussed in this analysis leads to several future research implications. Currently, there is much conflicting evidence in the HMB literature, with some studies showing an ergogenic effect, and others not. In this manuscript, we have written a qualitative analysis on why this may be the case; we believe the next logical step should be a quantitative review. The last meta-analysis to be conducted on HMB was over 6 years ago by Nissen and Sharp [41], and only 6 studies were examined. We suggest that a future meta-analysis pay close attention to various moderator variables including, exercise modality, training loads, training experience, age, and several dependent measures such as markers of muscle damage, strength, and DOMS. An additional analysis of the range of authors and Universities, which performed these studies would be helpful, to further address the issue of clustering and bias.

Current evidence suggests that increasing HMB dosages up to 3 grams will improve strength and lean body mass, and lower muscle damage in a dose dependent manner [36]. To date, only two studies [19,64] have investigated the efficacy of higher dosages of HMB (3 vs. 6 grams per day), with no additional benefits found with higher dosages, suggesting that 3 grams (or 38 mg/kg of body weight per day) is an optimal dosage for HMB. However, we propose that this optimal dosage may change with varying degrees of muscle damage and catabolic stimuli. As a purported agent capable of strengthening sarcolemal intregity and blunting proteolysis, HMB appears to exert its maximum effects during damaging and catabolic states induced by factors such as exercise [36], negative energy balance [51], and cancer [35]. Currently, no study has investigated the optimal dosage of HMB under varying degrees of catabolism. Two possible ways to test this would be to 1.) combine two catabolic stimuli (i.e. aging and a negative energy balance) and 2.) include varying degrees of damaging stimuli (i.e. 5 sets of squats vs. 10 sets). A lack of muscle sarcolemal disruption may partially explain conflicting results in advanced athletes, who are more resistant to muscle damage [123]. Therefore, future studies on advanced athletes are encouraged to incorporate a closely monitored, and relatively novel periodized strength program, designed to increase performance across conditions, and to cause significant muscle tissue damage. Further, chronic studies are rare in the literature, with few studies lasting longer than 4–8 weeks in duration [88]. Therefore, extended experiments are in need.

Two additional factors in optimal HMB administration concern nutrient timing and the effects of acute HMB administration. Recent evidence has suggested that various indexes of anabolism (e.g. protein synthesis) are greater when amino acids are consumed post exercise relative to rest [128], and pre-exercise relative to post exercise [129]. These results have been commonly attributed to enhanced blood flow and nutrient delivery to the muscles. Therefore, it would be of interest to see if these results with amino acids can be extended to HMB supplementation. Secondly HMB is generally administered greater than or equal to 2 weeks prior to examining its effects on indicators of muscle damage. We recently investigated the acute timing effects of HMB on maximal voluntary contraction (MVC) and visual analogue scale (VAS) determined soreness in 16 non-resistance trained men (18–28 yr) randomly assigned to HMB-PRE or HMB-POST groups. All subjects performed an eccentric damaging protocol (55 maximal eccentric unilateral knee extension/flexion contractions) on two separate occasions, performed on the dominant or non-dominant leg in a counter-balanced crossover design. HMB-PRE (N = 8) received 3 grams of HMB before and a placebo after exercise, or a placebo before and after exercise. HMB-POST (N = 8) received a placebo before and 3 grams of HMB after exercise, or a placebo before and after exercise. Tests for MVC and soreness were recorded prior to all the way up to 72 hours post exercise. While there was an overall reduction in MVC and increase in soreness in the quadriceps and hamstring following exercise, we found no acute or timing differences, suggesting acute HMB consumption may not influence muscle soreness and strength whether administered prior to or following exercise. It is important to note that unlike past studies we administered HMB only prior to or following exercise, with no loading period. However the acute timing effects on indirect markers of sarcolemal integrity remain to be analyzed, but are currently under investigation in our lab. Pathways which may be affected by the timing of acute HMB supplementation including mTOR and Ub-proteolytic pathways will also need to be investigated. Finally studies examining the timing of HMB over a chronic periods (e.g > 12 weeks) need further research.

The second purpose of this paper was to provide a comprehensive discussion on possible mechanisms, which HMB may operate through. Currently, the most readily discussed mechanism has been attributed to HMB as a precursor to the rate limiting enzyme to cholesterol synthesis HMG-coenzyme A reductase. This hypothesis suggests that HMB may provide readily available substrate for the synthesis of cholesterol needed to form and stabilize the sarcolemma [31,36]. Paradoxically, studies have found that HMB is associated with lowered total and LDL-C levels, but only in cases of hypercholesterolemia (i.e., > 200 mg/dl) [31,52]. While no complete explanation has been offered, these effects may be related to the inclusion of calcium during HMB supplementation (100–200 mg per g of HMB). Research indicates that as low as a gram calcium supplementation lowers serum cholesterol concentrations through increasing bile acid excretion, leading to increased use of endogenous cholesterol from the liver for regenerative processes [106]. Future studies should examine the effects of HMB on cholesterol, while controlling for calcium intake.

An increase in research has been directed towards possible proteolytic pathways HMB may operate through. Evidence from cachectic cancer studies suggests that HMB may inhibit the ubiquitin-proteasome proteolytic pathway responsible for the specific degradation of intracellular proteins. HMB may also directly stimulate protein synthesis, through an mTOR dependent mechanism. It would be of interest to see if the effects of HMB on the Ub/pathway and mTOR can be extended to the exercise domain. Future studies are therefore, encouraged to broaden the scope of dependent measurements taken.

## Conclusion

The first purpose of this paper was to provide an in depth and objective analysis of HMB research. While various studies analyzed in this manuscript support the efficacy of HMB as an effective ergogenic aid for athletes that decreases DOMS, markers of muscle damage, and body fat, while increasing various markers of performance, including LBM and strength in resistance trained athletes, and OBLA and VO2 peak in endurance trained athletes, a number of studies analyzed did not support the efficacy of HMB supplementation. The current authors suggest that these conflicting results may in part be attributed to the variability in humans, inadequate sample sizes, and methodological issues such as the specificity of testing conditions, cases of overtraining, elicitation of an inadequate training stimulus in experienced participants, limited dependent variables, and short duration experiments. Collectively, these results warrant further research on HMB supplementation while taking into account these various issues. Tables 1 and 2 summarize the results from the HMB literature.

**Table 1:** Studies Which Support the Efficacy of HMB supplementation in Varying Populations

Experiment	Participants	Dosage/Duration	Biochemistry	Performance	Body composition
Nissen et al. [36]	Untrained	0, 1.5, or 3 g/day for 7 weeks	CK & 3-MH decreased, dose dependent	Greater Total weight lifted, dose dependent	Greater LBM, dose dependent
Van Someren et al. [32, 37]	Untrained	3 grams of HMB and .3 grams of KIC, prior to a single bout eccentric exercise	CK down	Greater 1-RM bicep curl and ROM, lower DOMS	NR
Jowko et al. [18]	40 M, untrained	P, 3 gram HMB, HMB &creatine, or creatine	Only HMB lowered CK, urine urea nitrogen, and plasma urea	HMB & creatine additive effect on weight lifted	HMB & creatine additive effect on LBM
Gallagher et al. [19, 93]	37 M, untrained	P, 38 or 76 mg/kg for 8 weeks	CK, no effect on lipid profile, immune system, or renal function	Greater Isokinetic & Isometric torque, independent of dose.	Greater LBM, no effect on FML, independent of dose
Nissen et al. [28]	40 M, trained and untrained	P or 3 g/day for 4 weeks	NR	Greater bench press 1-RM	Increase in LBM and FML
Panton et al. [38]	36 F, 39 M, varying training experiences	P or 3 g/d for 4 weeks	NR	Greater upper body strength	Greater LBM & FML.
Thomson [39]	34 experienced weight lifters	P or 3 g/d for 9 weeks	NR	Greater leg extension strength	NR
Neighbors et al. [40]	Experienced collegiate football players	P or 3 g/d	NR	NR	Greater LBM and FML
Nissen & Sharp [41]	Meta-analysis, 9 studies	P or 3 g/day	NR	.28% greater weekly strength	1.4% greater weekly LBM
Nissen et al. [63]	37 F	P or 3 g/day for 4 weeks	No effect	Greater Bench Press 1-RM	Greater LBM
Vukovich and Geri [42], Vukovich and Adams [43]	8 experienced cyclists	3 g/day HMB, leucine, or P, 2 weeks for each supplement.	NR	HMB increased time to reach VO2 peak, and VO2 at OBLA.	NR
Knitter et al. [17]	16 F & M, experienced long distance runners	P or 3 g/day prior to 20 KM run.	Lower LDH and CK.	NR	NR
Byrd et al. [44]	28 active M	P or 3/g HMB or creatine daily prior to downhill run	NR	HMB lowered soreness.	NR
Vukovich et al. [29]	31 untrained M & F	P or 3 g/day for 8 weeks	NR	Greater upper and lower body strength	Greater FML, no effect on LBM.
Vukovich et al. [46]	31 elderly M & F	P or 3 g/day for 8 weeks	NR	Greater Leg strength	Greater FML, trend for LBM (P > .06)
Flakoll et al. [45]	50 elderly F	P or 2 g of HMB, 5 g of arginine, and 1.5 g of lysine daily.	Greater protein synthesis	Greater functional mobility, leg and handgrip strength.	Trend FML (P=.08)
Panton et al. [47]	35 elderly M & F	P or HMB for 8 weeks	NR	Greater Functional mobility	No effect
Soares et al. [48]	Adult mice	HMB prior to hind limb immobilization	NR	NR	Less fiber damage, greater fiber diameter.
Cohen [51]	Dieting humans, in negative energy balance	3 g/day of HMB	NR	NR	Greater maintenance LBM
Coelho and Carvalho [52]	12 elderly M	3 g/day of HMB for 4 weeks	Lower LDL-C	Greater weight lifting strength	Greater LBM

M = Male; F = Female; LBM = Lean body mass; P = Placebo; FML = Fat mass lost; CK = Creatine kinase; LDL-C = Low density lipoprotein cholesterol; NR = Not reported; 3-MH = 3-methylhistidine.

**Table 2:** Studies Which do not Support the Efficacy of HMB Supplementation in Varying Populations

Experiment	Participants	Dosage/Duration	Biochemistry	Performance	Body Composition
Kreider et al. [64]	40 experienced resistance trained M	0, 3, or 6 g/day for 4 weeks	No effect markers of muscle damage	No effect on strength	No effect on LBM or FM
Slater et al. [65]	Experienced resistance trained M	0, 3 g/day for 6 weeks	No effect markers of muscle damage	No effect on strength	No effect on LBM or FM
Paddon-Jones et al. [66], Jennifer et al. [67]	Untrained M	0, or 3 g/day, 6 days prior to a single bout eccentric exercise	NR	No effect on soreness, ROM, or elbow flexor strength	NR
O'Connor and Crowe [20]	Elite M rugby players	P, 3 g/day HMB, or creatine and HMB, during season.	NR	No effect on multistage fitness test or maximal cycle test	NR
Jack et al. [68]	Elite collegiate football players	0, 3 g/day for 4 weeks during football training	NR	No effect on weight lifting strength	No effect on body composition
Jay et al. [69]	26 elite collegiate football players	0, 3 g/day for 4 weeks during 10 day training camp	No effect markers of muscle damage	No effect on performance	No effect on LBM or FM
Kreider et al. [70]	Division 1-A College Football	0, 3 g/day during 4 weeks of resistance training	No effect markers of muscle damage	No effect on strength or sprint performance	No effect on LBM or FM

M = Male; F = Female; LBM = Lean body mass; P = Placebo; FM = Fat mass; NR = Not reported.

There is compelling evidence that HMB supplementation may be useful for clinical muscle wasting conditions including AIDS, cancer, bed-rest, and during periods of caloric deficits. HMB also appears to be safe, and may improve various markers of health, including blood pressure and LDL-cholesterol. When supplementing with HMB, current evidence suggests that 1 g of HMB should be consumed 3 times per day, for a total of 3 g of HMB daily (or 38 mg/kg of bodyweight). However, more studies are needed to determine the optimal dosage and frequency of HMB supplementation, and the overall efficacy of HMB supplementation as an ergogenic aid for athletes.

The second purpose of this paper was to provide an in depth analysis of possible mechanisms that HMB may exert its effects. Results from this review showed that HMB appears to primarily exert its effects through protective and anticatabolic mechanism. The prevailing explanation is the cholesterol synthesis hypothesis. However, recent studies have shown that HMB's anticatabolic effects are at least in part mediated by attenuation of the activation and increased gene expression of the ubiquitin-pathway. Furthermore, there is evidence that HMB may directly increase protein synthesis.

## Competing interests

The author(s) declare that they have no competing interests.
